# Supplementary material for: Effect modification of socioeconomic status in the association between contraception methods and couple’s education: A secondary analysis of PDHS 2017–18
Source: PLOS Glob Public Health. 2024 Jul 5;4(7):e0003424. doi: 10.1371/journal.pgph.0003424 (PMC11226098; doi:10.1371/journal.pgph.0003424)
Supplement: Attachment — Submitted filename: Rebuttal letter (Plos1).docx [file pgph.0003424.s001.docx]

Manuscript number: PGPH-D-23-01854

Effect modification of socioeconomic status in the association between contraception methods and couple’s education: a secondary analysis of PDHS 2017-18

PLos Global Public Health

Thank you for the opportunity to revise the referenced paper. We carefully considered the comments offered by the reviewers and explain below how we revised the paper based on those comments and recommendations. We greatly appreciate your time and effort to provide such insightful guidance.

Please address both editor and reviewer’s comments.

| S.No | Comments | | Response |  |
| --- | --- | --- | --- | --- |
|  | Reviewer 1 | |  |  |
| 1. | This study evaluates the association between a couple’s education level and their influence on their choice of contraception. In addition, the study also assessed the role of socioeconomic status in modifying the association between a couple’s education and contraception choice. Using PDHS 2017-18 data, the couple’s education status, preferences of contraceptive use, and wealth quintiles were analyzed through multinomial logistic regression after adjusting for other confounding factors. findings from this study revealed that out of the total sample of 14,368 women, 67.52% (n = 9701) were categorized as non-users, 23.55% (n = 3383) employed modern contraceptive methods, and 8.94% (n = 1284) used traditional contraceptive methods. Multivariable analysis showed that educated couples belonging to higher socioeconomic status (SES) had the highest adjusted odds ratio [7.66 (CI:4.89-11.96)] of using modern contraceptives as opposed to uneducated couples of low socioeconomic statuses. The odds of using modern contraceptives were higher amongst mothers with five or more children [8.55 (CI:7.09-10.31)] as compared to mothers with fewer children when adjusted for other covariates. | | Thank you so much for your remarks on the study findings. We appreciate your overall remarks on our manuscript, |  |
| 2. | Thus, this study concludes the dynamic interplay between a couple’s level of education, contraceptive preference, and socioeconomic status This study contributes valuable insights for the policymakers and stakeholders to understand the intricate relationship between these factors. | | Thank you again for your remarks on the study findings. We appreciate your overall remarks on our manuscript, |  |
| 3. | The strengths and limitations of the study should be highlighted. | | Thank you for your suggestions. Please review the strengths and limitations at the end of the discussion section. We had the strengths at the end of the discussion section. However, we have also written the limitations in the last paragraph of the discussion section. We are also copying the paragraph over here. “This study also has several limitations. Firstly, the dataset used is cross-sectional, which raises concerns regarding temporality and causality. These are inherent limitations of such designs, as they cannot establish causal relationships. Secondly, although we controlled for several potentially confounding variables, there remains a possibility of confounding due to other unidentified factors. Thirdly, residual confounding stemming from the variables included in our multivariable multinomial logistic regression analysis may also persist.” |  |
| 4. | Please review your reference list to ensure that it is complete and correct. If you have cited papers that have been retracted, please include the rationale for doing so in the manuscript text, or remove these references and replace them with relevant current references. Any changes to the reference list should be mentioned in the rebuttal letter that accompanies your revised manuscript. If you need to cite a retracted article, indicate the article’s retracted status in the References list and also include a citation and full reference for the retraction notice. | Thank you for the thoughtful comments and feedback. Following your guidance, we have reviewed and updated the reference list to ensure its completeness and accuracy. In our revised manuscript, the following updates have been made to the reference list:   1. **(Reference 5)** Nadeem, Muhammad, et al. "Women decision making autonomy as a facilitating factor for contraceptive use for family planning in Pakistan." Social Indicators Research 156 (2021): 71-89 2. **(Reference 6)** Zaidi, Batool, and Sabahat Hussain. "Reasons for low modern contraceptive use—Insights from Pakistan and neighboring countries." (2015) 3. **(Reference 7)** Sarfraz M, Hamid S, Kulane A, Jayasuriya R. ‘The wife should do as her husband advises’: Understanding factors influencing contraceptive use decision making among married Pakistani couples—Qualitative study. PLOS one. 2023 Feb 16;18(2):e0277173 4. **(Reference 8)** MacQuarrie KL, Aziz A. Women’s decision-making and contraceptive use in Pakistan: an analysis of Demographic and Health Survey data. Sexual and reproductive health matters. 2022 Jan 1;29(2):2020953 5. **(Reference 9)** Imran M, Yasmeen R. Barriers to family planning in Pakistan. Journal of Ayub Medical College Abbottabad. 2020 Oct 4;32(4):588-91 6. **(Reference 10)** Nishtar N, Sami N, Faruqi A, Khowaja S. Myths and fallacies about male contraceptive methods: a qualitative study amongst married youth in slums of Karachi, Pakistan. Global journal of health science. 2013 Mar;5(2):84. 7. **(Reference 19)** Asif MF, Pervaiz Z. Socio-demographic determinants of unmet need for family planning among married women in Pakistan. BMC public health. 2019 Dec;19(1):1-8 8. **(Reference 20)** Bashir S, Guzzo K. Women's Education, Spousal Agreement on Future Fertility Intentions, and Contraceptive Use in Pakistan. Studies in Family Planning. 2021 Sep;52(3):281-98 9. **(Reference 21)** Khalil B, Sarfraz M, Khan R. Socio-Economic and Demographic Determinants of Contraceptive Uptake in Pakistan. Pakistan Journal of Medical Research. 2015 Apr 1;54(2) 10. **(Reference 22)** Memon F, Jonker L. Educational level and family planning among Pakistani women: A prospective explorative knowledge, attitude and practice study. Middle East Fertility Society Journal. 2018 Dec 1;23(4):464-7 11. **(Reference 24)** Nasrullah M, Muazzam S, Bhutta ZA, Raj A. Girl child marriage and its effect on fertility in Pakistan: findings from Pakistan Demographic and Health Survey, 2006–2007. Maternal and child health journal. 2014 Apr;18:534-43 12. **(Reference 28)** Ali A, Zar A, Wadood A. Factors associated with modern contraceptive use among men in Pakistan: Evidence from Pakistan demographic and health survey 2017-18. Plos one. 2022 Sep 1;17(9):e0273907 13. **(Reference 30)** Slaymaker E, Scott RH, Palmer MJ, Palla L, Marston M, Gonsalves L, Say L, Wellings K. Trends in sexual activity and demand for and use of modern contraceptive methods in 74 countries: a retrospective analysis of nationally representative surveys. The Lancet Global Health. 2020 Apr 1;8(4):e567-79 14. **(Reference 31)** Bolarinwa OA, Tessema ZT, Frimpong JB, Seidu AA, Ahinkorah BO. Spatial distribution and factors associated with modern contraceptive use among women of reproductive age in Nigeria: A multilevel analysis. Plos one. 2021 Dec 8;16(12):e0258844 15. **(Reference 32)** Solanke BL. Factors influencing contraceptive use and non-use among women of advanced reproductive age in Nigeria. Journal of Health, Population and nutrition. 2017 Dec;36:1-4 16. **(Reference 35)** Hailegebreal S, Dileba Kale T, Gilano G, Haile Y, Endale Simegn A. Modern contraceptive use and associated factors among reproductive-age women in Ethiopia: multilevel analysis evidence from 2019 Ethiopia mini demographic and health survey. The Journal of Maternal-Fetal & Neonatal Medicine. 2023 Dec 15;36(2):2234067   We believe these revisions strengthen the credibility and relevance of our work. | | |
